# E-box binding transcription factors in cancer

**DOI:** 10.3389/fonc.2023.1223208

**Published:** 2023-08-03

**Authors:** Yuanzhong Pan, Pauline J. van der Watt, Steve A. Kay

**Affiliations:** ^1^ Department of Neurology, Keck School of Medicine, University of Southern California, Los Angeles, CA, United States; ^2^ Division of Medical Biochemistry and Structural Biology, Department of Integrative Biomedical Sciences, Faculty of Health Sciences, University of Cape Town, Cape Town, South Africa; ^3^ Institute of Infectious Disease and Molecular Medicine, University of Cape Town, Cape Town, South Africa

**Keywords:** E-box, circadian clock, cancer hallmarks, transcription factor, transcription control

## Abstract

E-boxes are important regulatory elements in the eukaryotic genome. Transcription factors can bind to E-boxes through their basic helix-loop-helix or zinc finger domain to regulate gene transcription. E-box-binding transcription factors (EBTFs) are important regulators of development and essential for physiological activities of the cell. The fundamental role of EBTFs in cancer has been highlighted by studies on the canonical oncogene MYC, yet many EBTFs exhibit common features, implying the existence of shared molecular principles of how they are involved in tumorigenesis. A comprehensive analysis of TFs that share the basic function of binding to E-boxes has been lacking. Here, we review the structure of EBTFs, their common features in regulating transcription, their physiological functions, and their mutual regulation. We also discuss their converging functions in cancer biology, their potential to be targeted as a regulatory network, and recent progress in drug development targeting these factors in cancer therapy.

## Introduction

An E-box is a regulatory motif of DNA, with the consensus sequence 5’-CANNTG-3’, that is found abundantly in most eukaryotic genomes. An E-box is a regulatory motif of DNA, with the consensus sequence 5’-CANNTG-3’, that is found abundantly in most eukaryotic genomes (see **Box 1**). Transcription factors (TFs) can bind to E-boxes in the promoter and enhancer region of genes through their basic helix-loop-helix (bHLH) domain or zinc finger domain to regulate their expression. E-box-binding TFs (EBTFs) regulate genes that are diverse in function. During development EBTFs determine the lineage commitment of skeletal muscle, cardiovascular and neuronal tissues, as well as hematopoiesis. In homeostasis they regulate many housekeeping genes and essential physiological processes, such as the cell cycle, circadian rhythm, and metabolism. Therefore, it is not surprising that EBTFs have fundamental functions in maintaining homeostasis and are deeply involved in tumorigenesis.

**Box 1 E-box overview.**

E-boxes were initially discovered in the promoter region of immunoglobulins to regulate their expression (1, 2), and later found to be widely present in eukaryotic genomes. The most prominent transcriptional regulators that bind E-boxes are the basic Helix-Loop-Helix (bHLH) proteins, which binds to the E-box through their bHLH domain (3). These proteins bind to DNA as hetero- or homodimers, in which the HLH parts interact to form dimers and the basic domain in the longer helix forms an α-helix to insert into the major groove of DNA to form a noncovalent bond. Some zinc finger domains can also bind to E-boxes, but the structural details are not clear. Distinct binding motifs of the proteins may have differential preferences to different variants of E-boxes (3). The binding specificity also depends on other factors such as their binding partner, flanking sequences near the E-box, and chromatin state (4, 5). Once a TF binds to an E-box, it can either activate or repress downstream gene transcription, depending on the cofactor and the cell context.

In homeostasis, E-boxes can be grouped by their binding TFs to regulate specialized physiological functions, such as MYC in the cell cycle, BMAL1 and CLOCK/NPAS2 in the circadian rhythm, HIFs in the hypoxia response, and EMT-TFs in embryonic development. These processes are delicately coordinated by their mutual regulations. One important node that connects their function is that these TF themselves are all regulated by E-boxes in their promoters. In addition, the turnover of cellular EBTFs is regulated by many shared mechanisms including transcriptional feedback control, miRNA control, and protein kinetics mediated by the ubiquitin-proteasomal system. The target genes and binding consequences of EBTFs in a tumorigenic context can be significantly different from those in a normal physiological background. This should be kept in mind when comparing and interpreting different studies on the EBTFs.

Another prominent function of EBTFs is their regulation of the development of specific tissues. Examples include heart and neural crest derivatives expressed (HAND) family TFs in the development of heart and lineage commitment of extraembryonic tissues; myoblast determination protein (MyoD) family factors in the differentiation of skeletal muscle tissues; neurogenic differentiation (NERUOD) family factors in neuronal tissue development; and T cell-acute leukemia protein (TAL1, also known as stem-cell leukemia factor, SCL) family factors in the regulation of hematopoiesis and angiogenesis. Since differentiation and proliferation potentials are in general considered mutually exclusive, these factors are generally not reported to be oncogenic.

Features of the promoters/enhancers in the genes that regulate developmental and essential cell activities have vastly different features, resulting from distinct motif components and binding factors (6). Thus E-boxes can be divided into at least two functional subgroups: those involved in tissue development and those involved in homeostasis maintenance.

Several families of EBTFs are widely studied in cancer biology. The MYC family of proteins feature prominently, as around 28% of tumors harbor at least one amplification of a MYC paralog, making it one of the most dysregulated oncogenic genes in human cancer (7). Hypoxia-inducible factor (HIF) proteins are also well known EBTFs, and the hypoxic hallmark of solid tumors has attracted much attention to this E-box-binding family of genes, which have also been shown to regulate many other processes in tumor development (8). Further EBTFs include those that regulate the epithelial-mesenchymal transition (EMT). It is generally accepted that these TFs are associated with stem cell features of cancer cells (9, 10). More lately, our lab and others have shown that the master circadian E-box-binding regulators, BMAL1 and CLOCK, also have important functions in several cancer types (11–14). These examples emphasize the importance of EBTFs in cancer biology.

Despite all the progress that has been made, the exact molecular roles of EBTFs in cancer is far from clear. In particular, a basic understanding of how these TFs select and participate in the transcription processes mediated by PolII is still lacking. In recent years, we have witnessed great progress in understanding the PolII transcriptional machinery in detail and the biology of regulatory elements in DNA. Such new knowledge provides unprecedented opportunities to rethink EBTFs in their most native role as DNA-binding proteins. By doing so we might be able to better understand this family of proteins and develop better strategies to target the TFs in cancer.

In this review, we review current understandings of the structure and molecular biology of the EBTF families that have been shown to play important roles in tumorigenesis. We discuss their mutual regulation to gain some insights into how these proteins are coordinated during tumorigenesis and tumor suppression, and we summarize the common processes they convergently regulate. At last, we propose that targeting the whole EBTF network in specific cancer types could be effective in suppressing multiple hallmarks of cancer simultaneously and have potential as a cancer therapeutic strategy.

## Important EBTFs in cancer

### MYC family proteins

**Figure 1:**
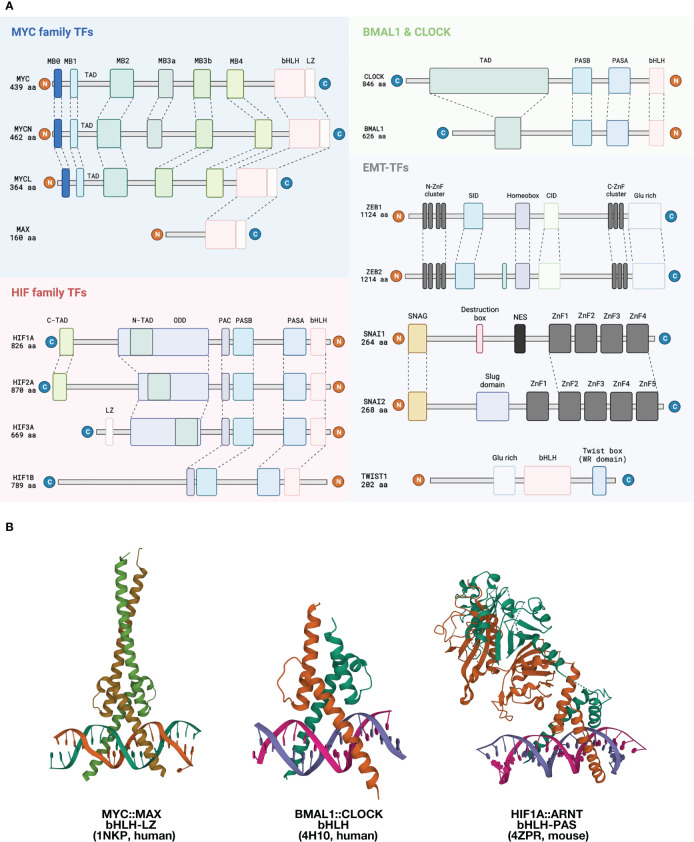
Structural overview of EBTFs. **(A)** Functional domains of EBTFs. Proteins can bind to E-boxes through a bHLH domain or a zinc finger domain. MYC family genes contain an extra leucine zipper domain downstream of bHLH. The three MYC family genes share highly conserved MYC-boxes (MB), except for MYCL lacking the MB3a. Other regions of the proteins have low degrees of conservation. MYC also contains a PEST domain (not shown) that might contribute to its fast turnover. MAX is a relatively small protein with a defined bHLH-LZ domain, but functions of the other regions of the protein are not well understood. Apart from MYC, MAX can also dimerize with itself or other bHLH TFs such as MAD and MLX. HIF family TFs feature the bHLH-PAS family TFs, which have a PAS-A and PAS-B domain immediately upstream of the bHLH. The PAS domain provides additional control of protein dimerization and might have a responsive function to environmental cues. A PAS-associated COOH-terminal (PAC) occurs C-terminal to the PAS motifs and is proposed to contribute to the PAS domain fold. The α-subunits of HIF proteins has an oxygen-dependent degradation (ODD) domain that contains two conserved prolyl residue (402 ODD and 564 ODD) that can be hydroxylated and induce proteasome-mediated degradation. They also have two transactivation domains (C-TAD and N-TAD) that facilitate target gene expression. BMAL1 and CLOCK are also bHLH-PAS family TFs, and BMAL1 has a defined TAD in the N-terminus. The ZEB and SNAI family bind E-boxes through their zinc fingers as monomers. ZEB1 and ZEB2 share up to 85% amino acid sequence homology in their zinc finger clusters but have low conservancy elsewhere. They both have a homeobox which seems to not bind DNA. Other defined domains are best known for interacting with other proteins. SNAI family proteins are featured by their conserved SNAG domain which is initially found in SNAI and GFI family proteins and a zinc finger cluster in the C-terminus. TWIST is a small bHLH protein that contains a characteristic TWIST box and binds to DNA as dimers. **(B)** Left: Structure of the bHLH-LZ domain of the MYC : MAX dimer binding to DNA (17). Middle: Structure of the bHLH domain of human BMAL : CLOCK dimer binding to DNA (18). Right: Structure of mouse HIF1A:ARNT bHLH-PAS domain dimer binding to DNA (19).

MYC was first discovered as a homolog of the viral oncogene *v-myc* in multiple chick retroviruses; thus the gene was named cellular-MYC (c-MYC) to specify its endogeneity. The MYC family has three members: the most prominent c-MYC, MYCN, which was initially found to be associated with neuroblastoma, and MYCL associated with small cell lung cancer, hence the names (15). The three MYC proteins have relatively limited sequence consensus, but they all share the entirely conserved bHLH-Leucine Zipper (bHLH-LZ) domain that binds to the E-box, and six highly conserved MYC boxes (MYCL lacks MB3a) that are known for interacting with other proteins (16). (**Figure 1A**, blue block) MYC will be used to refer to c-MYC in this article. Upon heterodimerization with its partner, such as MAX, MYC preferentially bind to the canonical E-box sequence 5’-CACGTG-3’ (20, 21).

The role of MYC in human tumorigenesis was exemplified by the translocated MYC coding sequences downstream from the immunoglobulin heavy chain enhancer in Burkitt lymphomas (22–24). Since then, MYC is found to be one of the most dysregulated, usually over-activated, oncogenic gene in human cancers (7). It is classified as a tumors-driving master transcription factor (MTF) in certain cancer types (25). Overexpression of MYC alone is sufficient to trigger a cancerous phenotypic change in cultured cells, and to induce *de novo* tumorigenesis in multiple mouse models (6, 26). The importance of MYC is also underscored by the fact that repression of MYC can result in fast regression of tumors in animal models, making it a promising target for tumor therapy (21). Despite its pivotal role in tumorigenesis and the great attention it attracted, the exact behavior of MYC is still far from clear.

The fundamental of MYC biology is that MYC function differs when expressed at high levels, as in many tumor cells, versus at relatively low physiological levels (16). MYC expression is ubiquitous but is delicately regulated to be kept at a low level in normal tissue. The turnover of MYC proteins is fast with a half-life of around 30 minutes (27). When in low abundance, MYC mostly binds to E-boxes and their close variants, whereas when overexpressed, it binds to more non-specific binding sites (28). This feature might be a result of the intrinsic disordered properties of the MYC protein, which allows it to dynamically interact with multiple partners in modest affinity. Therefore, in high concentrations, MYC specificity is easily overridden by a mass-action drive, leading to superfluous binding (20). The consequence of MYC binding is complex. There are currently several models describing the mechanism.

Classically, MYC is thought to be a pleiotropic transcription factor that activates, rather weakly, the transcription of genes through binding to the E-boxes in their promoters, as a heterodimer with its canonical cofactor MAX, which is also a bHLH-LZ protein (16). This model implies a group of “target genes” that are regulated by MYC. Attempts to recognize a set of MYC target genes using different large-scale analyses has resulted in sets with surprisingly small overlaps (16). This disparity might be a result of the abundance of E-boxes in the genome and their different open states in different cells, since MYC is generally considered a non-pioneer transcription factor, meaning that it only binds to chromatin regions that are already accessible but cannot open a closed chromatin, as was clearly shown in iPSC studies (29). To some extent, there is consensus over MYC target genes, including the HALLMARK MYC Target gene sets proposed by *Liberzon et al.* in the molecular signature database (MSigDB) (30). Such core common target gene sets have been useful as indicators of MYC activity in hypotheses-generating cases, but they should not be used to preclude potential genes regulated by MYC, especially in cancerous contexts where chromatin accessibility is largely remodeled and mutations in regulatory elements are common, resulting in *de novo* binding sites of TFs.

Apart from being an activator, MYC has also been proposed as a repressor of gene expression (31). The most studied repressive mechanism of MYC is through its interaction with two other proteins, MIZ1 (32) and SP1 (33), to recruit co-repressors. It has been proposed that the repressive function of MYC is of comparable importance to the activating function (34), although genomic-level correlation analysis implies rather weak effects of the repressive function of TFs in general (35). Finally, it is noteworthy that MYC can also regulate RNAPlI and RNAPIII-mediated transcription of ribosomal RNA and tRNA (36, 37), but this function is out of the scope of this review.

As a weak activator model is insufficient to explain its broad participation in various physiological processes of the cell and its strong tumorigenic effect, later a “general gene amplifier” model was proposed by two simultaneous papers and introduced a new view of MYC (38, 39). According to this model, MYC can act as an amplifier that increases the overall RNA production of the whole cell. This model can explain some observations in MYC-driven tumorigenesis, but still oversimplifies MYC function since elevated RNA production is neither sufficient nor necessary for tumorigenesis, and it fails to explain the complicated up- and down-regulation of genes after its levels change.

Given the different behaviors of MYC at distinct levels in the cell, a gene-specific affinity model has also been proposed (16, 40). According to this model, promoters of different genes require different levels of MYC protein to activate their transcription. This is possible due to the relatively low affinity of MYC-MAX binding. This model provides an explanation of the paradox of broad DNA-binding and specific gene regulation by MYC, but still lacks the ability to unify MYC function in different tumors and ignores the broad involvement of MYC in multiple processes of RNAPII-mediated transcription.

Recent progress on characterizing the protein interactome of MYC has shed new lights on understanding the function of MYC and further expands the role of MYC function in cancer. The interactome of MYC was revealed by mass-spectrometry analysis of immunoprecipitated MYC or through BioID screening (41, 42). Functional assays combined with selective depletion of certain MYC boxes has also revealed specific functions of different MYC boxes. These studies have helped to define a core group of MYC-associated proteins (16, 43). These MYC interactors mark the broadness of MYC function since they are involved in various fundamental cellular processes, such as chromatin topology and remodeling, the cell cycle, general transcription, and ubiquitination. The interactome also reveals more fundamental functions of MYC in participating in general transcription mechanisms, including the formation of the preinitiation complex, initiation, pausing, elongation, and splicing (43). This aspect of MYC function reinforces the essentiality of MYC in tumorigenesis (16).

These newly revealed mechanisms urge that more basic structural understanding and regulatory element logic are key to dissect the role of MYC in cancer (16).

### Hypoxia-inducible Factors

The hypoxia-inducible factor (HIF) family TFs exemplify the bHLH-PAS family of proteins. In general, these proteins are characterized by a PER-ARNT-SIM (PAS) domain, which contains PAS-A and PAS-B located instantly upstream the bHLH domain (44, 45). (**Figure 1A**, pink block) Similar to MYC and MAX, bHLH-PAS proteins also form heterodimers, with an α-subunit serving as a stimuli-responder or regulator of tissue specificity and the β-subunit expressed more stably and ubiquitously. The PAS domain serves as another layer of dimerization control on top of bHLH for higher specificity, and the PAS-B domain can sometimes serve as a sensing domain that can bind to small molecules in the environment or sensory/regulatory proteins (46). bHLH-PAS TFs have a more defined structure than MYC, which is rather disordered (**Box 2**). In the case of HIF proteins, there are three α-subunits, HIF1, 2, and 3-α. They can all dimerize with the ubiquitous β-subunit HIF1β (also known as ARNT) (55). Dimerization determines the binding to specific E-box variants. The consensus binding motif of the HIFα-ARNT dimer is 5’-A/G-CGTG-3’, which is an E-box variant usually referred to as the hypoxia responsive element (HRE) (55). HIFs mainly serve as activators of gene transcription once bound to DNA. A hypoxia ancillary sequence 5’-CAGGT-3’ that is located only several nucleotides downstream from the HRE has been proposed to be necessary for HIF activation of VEGF and EPO – two well-documented HIF-target genes – but this sequence lacks the structural basis for HIF to bind and is likely to be dispensable for other genes (56).

**Box 2 Structural insights of bHLH dimerization and DNA-binding.**

The bHLH domain is a highly abundant DNA-binding domain present in a large number of human proteins. Some bHLH domains co-occur with a leucine-zipper motif, such as that present in MYC and the components in its close regulatory network. These are termed bHLH-LZ proteins. Another important family is the bHLH-PAS TFs in which the tandem PAS-A and PAS-B domains that are located immediately upstream bHLH. All bHLH family proteins bind to DNA as dimers. [figures are adapted from ref. (17) and ref. (19)].**DNA-binding and dimerization features of the bHLH-LZ-containing MYC and MAX:** MYC was first found to bind the canonical E-box in a dimer (47). However, MYC alone cannot form a DNA-binding dimer, and this entailed efforts to search for its binding partner, leading to the discovery of MAX (48). The inability of MYC to form homodimer is a result of its LZ region, which exhibits minimal helicity (49). In a putative homodimer of MYC, this region forms a helix that has residues Glu410, Glu417, and Arg424 at the interacting interface of the coil structure that would repel each other through interfacial electrostatic force and destabilizing the putative dimer (50, 51). On the contrary, MAX can form homodimers and bind to DNA (50). MAX dimer does not have the repelling residues that precludes homodimerization, but harbors residues that weaken the affinity (17, 52). These residues become complementary in the MYC : MAX dimer, making the heterodimerization of MYC and MAX the favorable form of existence in the nucleus (17). (see the figure, part A) Upon dimerization, MYC and MAX undergo structural changes and gain a more defined secondary structure, with MYC forming an additional quaternary structure, as shown by circular dichroism (53).

Specificity of the MYC : MAX dimer binding to the canonical E-box sequence 5’-CACGTG-3’ is mediated by the conserved His359/Glu363/Arg367 motif in MYC (17). His359 forms a H-bond with the central guanine and lead to the specificity for a purine; Glu363 makes H-bonds with the adenine and the cytosine at position 2 and 3; and Arg 367 forms a H-bond with the guanine in the center and with the phosphate group between the cytosine and adenine at position one and two. Together these residues control the preference of the MYC : MAX dimer towards the 5’-CACGTG-3’version of E-box (17). The heterodimer also has additional contacting sites with the phosphate backbone at Lys371 and Lys389 of MYC and equivalent positions of MAX, which are located in helix 1 and in the loop, respectively (17). Other bHLH-containing proteins with a hydrophobic residue at 367-equivalent position preferentially bind to a Type A E-box 5’-CAGCTG-3’ (17).

**Structural features of the bHLH-PAS-containing BMAL1:CLOCK and HIF1A:ARNT:** bHLH-PAS is a highly conserved motif series that determines protein dimerization, and bHLH-PAS-containing proteins share certain degrees of structural similarity within and across families. The HIF family proteins HIF1A and HIF2A both dimerize with ARNT and the bHLH-PAS domain structure of both dimers are very similar (19). On the other hand, both BMAL1:CLOCK and HIF1A:ARNT dimers feature close interactions in all three domains (bHLH, PAS-A, and PAS-B) with an asymmetric arrangement of the two molecules (19, 54). The interacting modes at the three domains are also similar. The bHLH domain forms a four-helical bundle, which is also observed for other bHLH TFs such as MYC : MAX and USF. The PAS-A domains interact with each other through contacts between the α-helix of one molecule and the β-sheet concave surface of the other molecule. (see the figure, part B) In addition, ARNT and BMAL1 share identical core residues (Arg102, Glu98, and His94) that recognize the 5’-GTG-3’ half site of the E-box (18, 19, 54).

Meanwhile, the PAS domains of the two families also exhibit characteristic differences. Prominently, the PAS-A and PAS-B domain of BMAL1 have a connected surface, whereas the two domains are displaced in ARNT (19, 54). (see the figure, part C) This feature of ARNT might be the reason for its ability to dimerize with many other bHLH-PAS members, including HIF1-3A, NPAS1,3-4, AHR and SIM1-2, while BMAL1 has only been shown to dimerize with CLOCK and NPAS2.

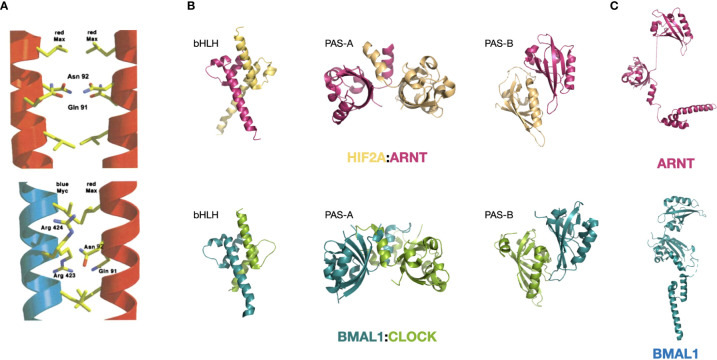

In normoxic conditions, HIF-1α is continuously expressed but undergoes fast hydroxylation mediated by prolyl-4-hydroxylase (PHD) at conserved proline residues. Hydroxylated HIFs will bind to the von Hippel Lindau (VHL) E3 ligase and be polyubiquitinated, then undergo degradation in the proteasome. As oxygen level goes down, HIF α-units are stabilized and dimerize with the β-unit, then bind to DNA and activate the transcription of target genes. Gene activation via HIF1A/2A is associated with two transactivation domains (TADs), with the N-TAD located in the oxygen-dependent degradation domain and the C-TAD at the C-terminus. The C-TAD domain interacts with CBP and p300, recruiting them to the HRE motif of target genes, which modify the local chromatin, and interact with the core transcription machinery to activate gene transcription. Other cofactors of HIF1α/2α include PKM2 (which builds a direct link to the Warburg effect), and a CDK8-mediator which promotes pause release of RNAPII (57, 58). It is noteworthy that ARNT itself can act as a coactivator of other factors without HIF-α (59). Multiple studies have attempted to identify a set of HIF target genes, using both experimental and computational methods (60, 61). Similar to MYC, and potentially for the same reason, efforts to define target genes of HIFs in different types of cells has resulted in a small intersection set which can serve as a core group of HIF-regulated genes.

The most prominent role of HIF in cancer is its regulation of metabolism in response to the hypoxic tumor microenvironment. But like MYC and other EBTFs, HIFs are also found to be involved in many other aspects of tumorigenesis, including angiogenesis, the immune response, epigenetic regulation, the epithelial-mesenchymal transition (EMT), etc. An example of HIF as a key driver of tumorigenesis is in clear cell renal cell carcinoma (ccRCC), where mutation in VHL is observed in most cases and leads to aberrant accumulation of HIFs (62). HIF1 and HIF2 are both involved in this type of cancer and have complicated interactions, exemplifying how EBTFs from the same family can coordinate to fuel tumorigenesis.

### BMAL1 and CLOCK circadian clock proteins

The core mammalian circadian regulators BMAL1 and CLOCK are another example of bHLH-PAS proteins. (**Figure 1A**, green block) The level of BMAL1 mRNA and protein oscillates with an approximate 24-hour period as a result of a tightly regulated feedback loop, whereas CLOCK levels stay relatively stable. BMAL1 and CLOCK form a heterodimer and bind to the canonical E-box sequence to activate the transcription of target genes, including their own repressors such as the cryptochromes CRY1/2 and the period genes PER1/2/3. PER and CRY proteins can form a complex to repress the transcription mediated by BMAL1-CLOCK, thus forming a negative feedback loop to induce circadian oscillation of gene expression. Two additional layers of feedback control of BMAL1-CLOCK function exist, mediated by the nuclear receptors REV-ERBα, REV-ERBβ and ROR, which make up a tripartite feedback mechanism of circadian gene expression, which is reviewed in detail by *Takahashi* (63).

In mice, the BMAL1-CLOCK dimer activates transcription of target genes in the morning. Then as the PER and CRY protein levels accumulate in the late afternoon, they translocate into the nucleus to interact with BMAL1-CLOCK to repress the transcription mediated by the dimer. PER and CRY are then targeted and degraded by proteasome, leading to reactivated BMAL1 and CLOCK to start a new transcription cycle in the morning (63). BMAL1-CLOCK activation involves chromatin interaction and modification. Like HIFs, BMAL1 and CLOCK also interact with p300 and CBP to acetylate histones for transcription. CLOCK itself has been shown to have histone acetyltransferase activity and can acetylate H3K9 and H3K14 (64).

Unlike MYC and HIF, BMAL1-CLOCK has been proposed to have pioneer properties and can open closed chromatins (65). But in a physiological context, this function seems to have specific requisites for certain cofactors, which can be tissue-specific (66), therefore the actual binding sites of physiological BMAL1-CLOCK still depend on specific contexts, such as in different organs. Again, defining a set of BMAL1-CLOCK target genes by intersecting sets in different contexts results in a small group of genes primarily regulated by BMAL1 and CLOCK (67). This gene set is sometimes referred to as clock-controlled genes (CCGs), but note that CCGs are defined by their 24-hour rhythmic expressions and comprise different genes depending on the context in which they are defined.

A disrupted circadian rhythm at an organismal level has long been marked as a potential risk factor for cancer. However, the molecular function of BMAL1 and CLOCK in tumors has only been studied rather recently. Part of the reason is that mutations in BMAL1 and CLOCK are not commonly observed in cancer, implying that they themselves do not commonly function as mutated drivers of tumor initiation. Nonetheless, BMAL1, CLOCK, and other core clock genes have been shown to be widely dysregulated at the transcriptional level across cancer types (68). This is also true for MYC and HIFs, implying transcriptional mechanism of driving tumorigenesis might exist.

Recent studies have discovered pivotal roles for BMAL1 and CLOCK in multiple types of cancers. For example, in glioblastoma (GBM) stem cells, acute myeloid leukemia (AML), and hepatocellular carcinoma (HCC), BMAL1 is essential for the proliferation of tumor cells (12, 13). Knock-down of BMAL1 can significantly reduce the growth of tumors both *in vitro* and *in vivo.* In GBM, BMAL1 has been shown to gain thousands of new binding sites compared to normal neural stem cells and is rewired to support tumor specific metabolism of both glucose and fatty acids (13). These results echo the case of MYC where EBTF functions vary significantly between tumorous and physiological conditions.

It is noteworthy that, at the cellular level, malignancy does not necessarily disrupt the circadian rhythm of the cell, since cancer cells can either have strong circadian rhythms or be totally arhythmic (13, 69). We recommend that in a tumorigenic context, distinctions should be made between the function of the circadian TFs in tumor cells and the actual circadian rhythm of cells and organisms.

## EMT transcription factors

Importantly, the key transcription factors governing the process of epithelial-mesenchymal transition (EMT) are all E-box binding proteins. EMT was first discovered as an essential process during certain stages of embryo development such as gastrulation (70). In cancer cells EMT is featured by upregulation of mesenchymal markers such as vimentin (VIM), and downregulation of epithelial markers such as E-cadherin. EMT in cancer was initially studied in relation to its role in metastasis (71). Although a wide consensus on EMT has not been reached yet (10, 71, 72),now it becomes generally accepted that tumor cells can have intermediate hybrid E/M states spanning a continuous E-M spectrum, and a more hybrid state is associated with more aggressive stem cell properties (72–75).

Multiple families of TFs can induce EMT in cancer, including the SNAIL family, SNAI1 and SNAI2, bHLH-containing proteins TWIST1 and TWIST2, and the zinc-finger E-box binding homeobox family, ZEB1 and ZEB2. They all bind to E-boxes to induce an EMT program in cells, but the specific functions of different proteins are non-redundant (75). SNAI1 has a zinc finger domain that consists of four zinc finger motifs and can bind to the E-box variant 5’-CAGGTG-3’. The SNAG domain can compete with H3 to prevent lysine-9 from being demethylated, hence activating gene expression (76). The ZEB proteins have two zinc finger clusters that bind to 5’-CAGGTG/A-3’ and show higher affinity to promoters that have two E-boxes with variable distances in between, such as in the case of CDH1 (77). TWIST binds to the E-box as a homo- or heterodimer and can act as both a repressor and activator of gene transcription. The different binding preferences of the TFs forms the basis of their distinct functions (77).

EMT-TFs can regulate the expression of a set of common genes and their own specific targets as either repressors or activators. Their most prominent common function is to repress the expression of CDH1 through binding to the E-boxes in the promoter region of the gene. Other common target genes include the interleukins and TGF-β superfamily genes. Currently the most prominent function of the EMT-TFs is their regulation of cancer stem cell-related features such as drug resistance, phenotypic plasticity, immune evasion, etc. (75) Importantly, EMT-TFs can function in non-epithelial types of cancer such as glioblastoma (78). Therefore, researchers have suggested that instead of focusing solely on the EMT program, more attention should be placed in understanding the specific functions of the different EMT-TFs (9).

## Other E-box binding TFs reported in cancer

Upstream stimulatory factors (USF) 1 and USF2 are ubiquitously expressed transcription factors that both have a bHLH-LZ domain that binds to the E-box as a heterodimer or homodimer (79). They also contain a USF-specific region (USR) upstream of bHLH that is important for E-box dependent transactivation. USFs are transcription activators that have a small group of defined target genes. USF1 is associated with familial combined hyperlipidemia and was found to bind the promoter of genes that regulate lipid and cholesterol metabolism, which is often dysregulated in cancer cells (80). USF2 can compete with MYC to antagonize the function of MYC (81). USF1 also interacts with p53 and regulates its function (82).

Other bHLH-PAS proteins such as AHR and NPAS in cancer are reviewed in ref (46).

## Regulatory features of E-box-containing regulatory elements

**Figure 2:**
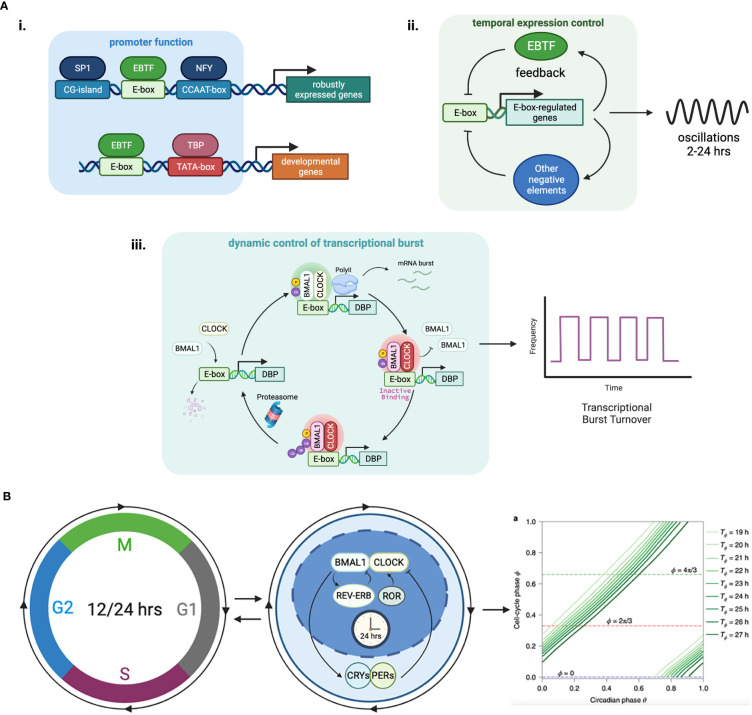
Functional features of E-box elements in transcriptional regulation. **(A)** (i) E-boxes are used to generate oscillations of gene expression through negative feedback loops. The negative regulator in the loop can be an EBTF itself, such as in the case of HES1 which features a 2-hour ultradian rhythm, or target genes of EBTFs, such as PER and CRY in the case of BMAL1, which generates a 24-hour rhythm. ii. E-boxes determine different functions of the promoter by working with different partner motifs. For example, in TATA-less promoters that have high CG-content, E-boxes participate in keeping the genes continuously/robustly expressed, whereas in some developmental genes an E-box works together with the TATA-box to initiate downstream developmental programs. iii. EBTFs can serve as a temporal control node in the turnover of transcription burst. The physiological function of such control is unclear. **(B)** Phase lock of circadian clock and cell cycle. Mathematical modeling and experimental validation revealed that the two oscillators of circadian rhythm and cell cycle exhibit multiple phase-locked states that exhibit robustness against molecular fluctuations.

Although EBTF families feature distinct functions, certain features of E-box-regulated genes are commonly observed. Some features of regulatory elements in promoters are discussed in **Box 3**. In an early study that analyzed the promoters of CCGs, it was found that some other motifs are overrepresented in addition to E-boxes, including those of SP1, ZF5, NRF1, and EGR, which represent CG-rich motifs (67). This implies that EBTFs might recruit general TFs under the assistance of SP1. Other overrepresented motifs include NFY and E2F family factors (67). In mechanistic study on the EBTF sterol regulatory element-binding protein (SREBP) family member SREBP1, SP1 and NFY are reported to be partner factors (84). Interestingly, in an analysis that aimed to identify overrepresented motifs in bidirectional promoters (defined as promoters of less than 1kb length and flanked by two protein-coding genes that are transcribed in opposite directions), E-box, E2F, NRF1, NFY, and CG-rich motifs were also reported (85). These reports emphasize the importance of co-motifs in determining the function of E-boxes. Conversely, the TATA-box is usually absent in these bidirectional promoters as well as housekeeping genes (85). Although the TATA-box and E-box are not mutually exclusive in promoters, genes containing both motifs implement highly specialized functions such as regulating certain developmental programs (86, 87) (**Figure 2Ai**). As any functional promoters or enhancers comprise multiple TF-binding sites, these features imply that E-boxes and other motifs have specific functions in regulating transcription and their distinct combinations encode specific types of transcriptional regulation.

**Box 3 Functional features of regulatory elements.**

Regulatory elements of the genome (enhancers and promoters, etc.) encode the information for spatial and temporal regulation of gene expression. TF-binding motifs are the building blocks of such information and determine the structure of the genome, epigenetic states of the chromatin, and the RNA polymerase-mediated transcription. A promoter region is loosely defined as the region upstream of the TSS that is several hundreds of base pair long. The short region up to 100 bp flanking the TSS is called the core promoter, which is sufficient for the assembly of the pre-initiation complex (PIC). The farther part of the promoter is termed the proximal promoter, which is usually bound by TFs to facilitate transcription and has enhancer activity (83).

Some patterns of promoter components and functions have been recurrently observed. The first type of promoters which is often referred to as ‘focused’ or ‘sharp’ promoter features a focused initiation site and is usually found in tissue- or cell-specific genes (6). Focused promoters often contain a eukaryotic core promoter motif such as TATA-box and initiator motifs and lack CG-rich elements. On the contrary, some promoters have multiple closely concatenated TSSs, the uses of which are observed in similar frequency. This type of dispersed promoters is prominently found in housekeeping genes and often have CpG motifs. These promoter types also share specific features of histone modifications, which is discussed in detail in ref (83).

These two types of promoters exemplify the connection between the promoter functions and components. For example, CG-rich motifs are inherently nucleosome-repelling, and their existence might improve the robustness of the promoter to maintain the expression level of essential genes. These properties of regulatory elements form the theoretical basis that TFs binding to the same class of motifs share functional features, and these features are defined by the combination pattern of partner regulatory motifs.

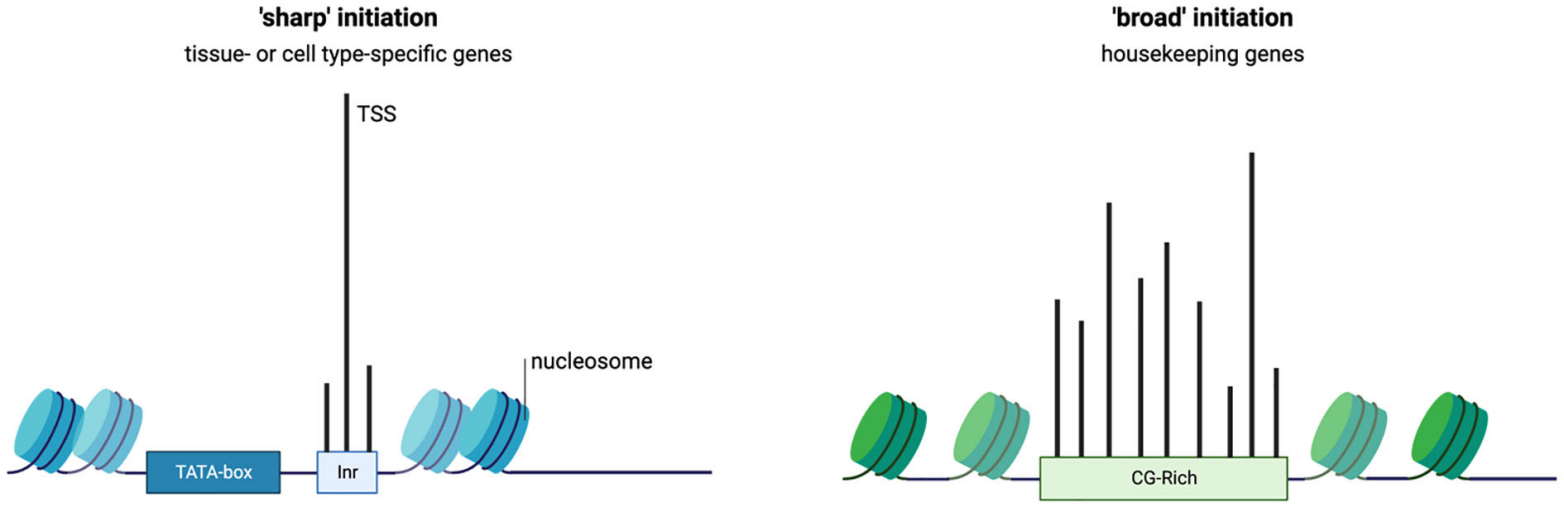

Little is known about the transcriptional regulatory properties of E-boxes. One of their important functions is to convergently coordinate the temporal control of gene expression through oscillators (**Figure 2Aii**). Oscillator is a general mechanism of dynamic transcriptional control that can be achieved by feedback loops (88, 89). The most prominent example is the 24-hour circadian rhythm implemented by the tripartite feedback loop as described above. The circadian clock is intimately interlocked with the cell cycle, which is another oscillator that is dominated by the dynamics of MYC. The molecular details of the coupling of these two oscillators and their dynamics and system properties are extensively studied with experiments and mathematical modeling (90–94) (**Figure 2B**). Because EBTFs are usually regulated by E-boxes too, they can form interlocked feedbacks and provides a space for temporal control of various period lengths. The bHLH TF HES1 exemplifies a 2-hour ultradian oscillator through a self-feedback loop via an E-box in its promoter (95). The core inflammatory TF NF-κB is also an example of intrinsic oscillatory gene regulated by EBTFs (96).

Another feature of EBTFs including MYC and BMAL1 is they fit in the Kamikaze model of transcription, where ubiquitin-dependent proteolysis is required for RNAPII elongation, and newly synthesized activators need to be loaded for a new round of transcription burst (97–99). This feature might serve as a mechanism driving the temporal control of cell activities by EBTFs, but the molecular details and the kinetics of this type of transcription is largely unknown (**Figure 2Aiii**).

Another key function of E-boxes might be to maintain the robustness of expression of essential genes, since many of the genes containing E-boxes are ubiquitously expressed and regulate basic physiological activities of the cell. Supporting this hypothesis, genes driven by E-box-enriched bidirectional promoters are expressed in a higher frequency than the average of all human genes (85). Furthermore, in *Drosophila* where CpG islands are absent in promoters, E-boxes are often found in promoters of housekeeping genes (100). This intrinsic robustness of E-box-containing genes underlies the importance of E-boxes in cell homeostasis (100) (**Figure 2Ai**).

In recent years our understanding of the PolII-mediated gene transcription mechanism has been greatly expanded upon, and its role as a potential therapeutic target in cancer has been explored (101–103). This progress has also revealed new functions of EBTFs in regulating multiple steps of transcription. MYC is a prominent example. Firstly, evidence has shown that MYC can facilitate the formation of the preinitiation complex of PolII by interacting with TATA-box binding protein (TBP) and potentially modulating the energetic landscape of TFIID during preinitiation complex (PIC) formation (104). GTF2F1, which is a component of TFIIF, binds directly to MYC through MB0 and serves another way through which MYC participates in PIC assembly (42). During initial PolII elongation, MYC has been shown to facilitate mRNA capping by recruiting RNGTT and RNMT (105). During productive elongation, MYC can recruit positive transcription elongation factor b (p-TEFb) and enable CDK9 to phosphorylate Ser2 on the CTD of PolII and allow PolII to continue with productive elongation (106, 107). A recent report showed that SPT5, another key regulator of elongation, is recruited by MYC (41). Other EBTFs have also been reported to participate in these steps of RNAPII transcription.

## Mutual regulation of different EBTFs in cancer

**Figure 3:**
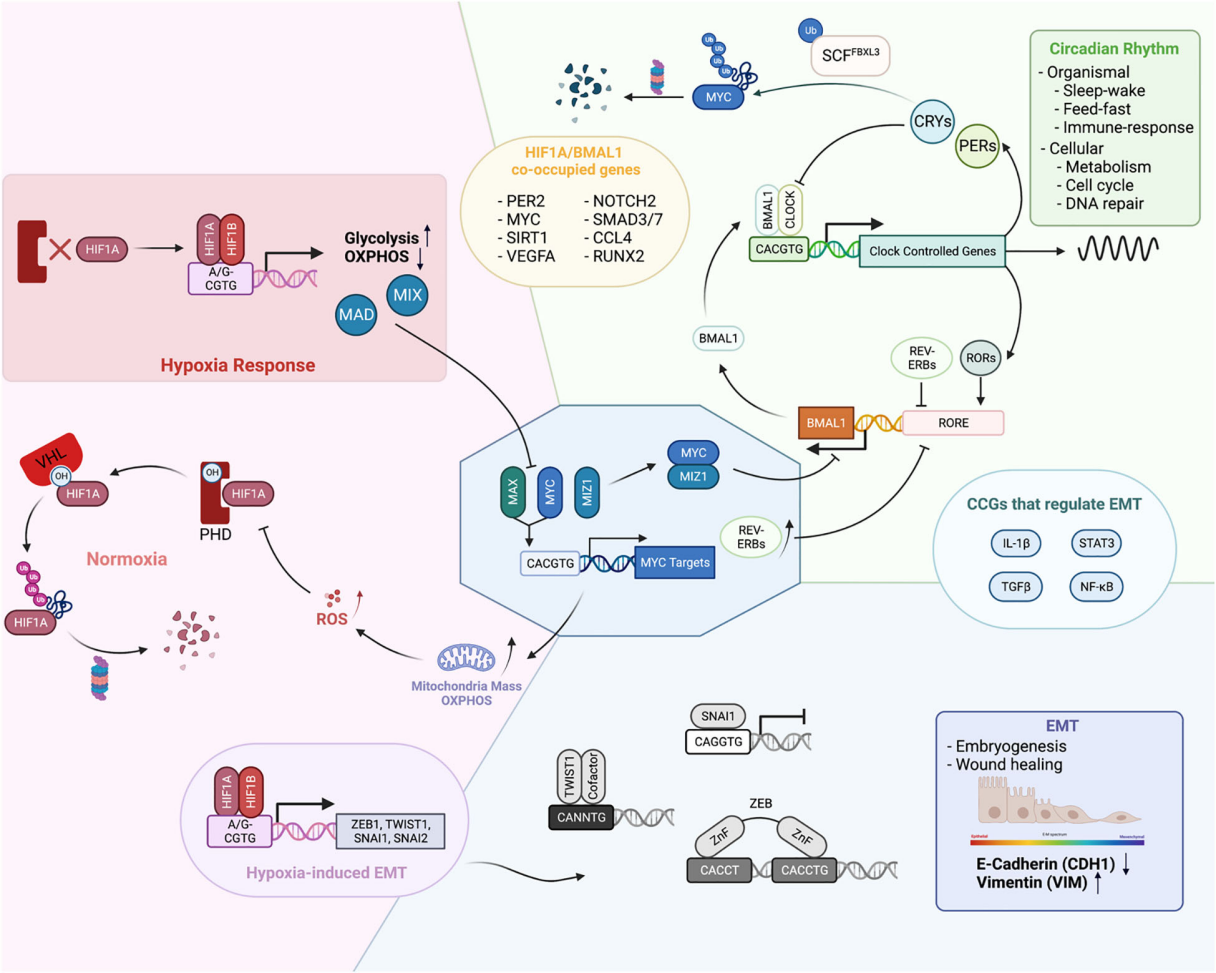
Physiological functions of EBTFs and their mutual regulation to form a coordinated network. **(A)** In homeostasis EBTFs each have their prominent functions, including control of the cell cycle by MYC, oxygen sensing by HIF, and control of the circadian rhythm by BMAL1 and CLOCK. The colored blocks highlight the primary physiological function of each EBTF family. MYC tightly regulates the expression of E2F target genes to control the cell cycle. HIF is continuously expressed in cells and rapidly degraded by the proteasome under normal oxygen and ROS levels. When oxygen levels are low and HIF proteins are less hydroxylated, they are stabilized and bind to DNA to activate downstream gene expression. BMAL1 and CLOCK dimerize and bind to DNA to activate clock-controlled genes, which include their own suppressor CRYs and PERs. CRY and PER translocate into the nucleus and form a repressive complex to inhibit BMAL1 and CLOCK transcriptional activity, forming a delayed negative feedback loop. When they are degraded, a new cycle of BMAL1 and CLOCK transcriptional activity is activated. Reported mutual regulations across different EBTFs are exemplified at the interface of each color block corresponding to the two families. As they bind to the same DNA motif, regulate each other, and contain E-boxes in their own promoters, their functions unavoidably converge into a network to coordinate the essential processes of the cell.

As EBTFs all bind to E-boxes and share common regulatory features, it is not surprising that they can mutually regulate each other, both within each protein family and across different protein families (**Figure 3**). This can occur via competitive binding to DNA, direct binding to each other, or regulation of the turnover of each other. These interactions connect EBTFs into a dense regulatory network.

### MYC-HIF interactions

MYC and HIF closely interact with each other. HIF1α can inhibit MYC activity through various mechanisms. It can not only bind directly to MAX and interfere with MYC-MAX dimer activity, but also activate expression of other MYC competitors such as MAD and MXI1 (108). HIF1α also competes with MYC for DNA binding, which is exemplified in the promoter region of p21 (109). Additionally, HIF1α has been reported to promote the proteasomal degradation of MYC (110). Paradoxically, MYC has been mostly reported to promote the activity of HIF1α. This is exemplified by decreased HIF1α levels after MYC knock-down in multiple myeloma cells, and stabilization of HIF1α proteins after MYC overexpression (111). Mechanistically, MYC can reduce the binding of HIF1α to the VHL complex and decrease its degradation (112). In addition, MYC increases mitochondrial OXPHOS and ROS production, which inhibits PHD activity in non-hypoxic conditions (113). The paradox also lies in the functional consequence of normal MYC and HIF1α activity. MYC usually promotes the function and biogenesis of mitochondria, whereas HIF1α represses them by activating FOXO3a which consequently represses mitochondrial gene expression and induces BNIP3, triggering mitochondria degradation through autophagy (114).

However, in cancer cells MYC and HIF1α are not incompatible since many cancer types have both MYC and HIF1α in high levels. The seeming conflict can be explained by the deregulation of MYC levels. When at high levels, MYC can still maintain its activity stoichiometrically and override the inhibitory effect of HIF1α (115). USP29 has been shown to maintain HIF1α and MYC levels at the same time to promote tumorigenic metabolism (116). In such cases, MYC and HIF1α may cooperatively tailor a gene expression program that takes advantage of the pro-tumorigenic aspect of each protein to fuel tumor growth. For example, both proteins activate genes for glucose import and glycolysis such as LDHA and HKII, which contributes to the Warburg effect in cancer (117). This exemplifies how EBTFs coordinate in a network to fuel cancer progression.

Such cooperation is also observed for HIF2α, although unlike HIF1α, HIF2α is better known to promote MYC activity in cancer. HIF2α enhances MYC function by stabilizing the MYC-MAX dimer in clear cell renal carcinoma cells and colorectal carcinoma cell lines (118). In turn, MYC has been reported to activate HIF2α transcription by directly binding to its promoter in T cell leukemia and maintaining a pool of cancer stem cells (119). By contrast, in physiological endothelial cells, it is reported that HIF2α represses MYC expression (120).

### MYC-BMAL1/CLOCK interactions

The circadian clock proteins also closely interact with MYC through various mechanisms. Two groups have shown that MYC (both MYC and MYCN) can inhibit BMAL1 and CLOCK function in cancer cells and disrupt the circadian dynamics of the core clock molecules (121, 122). Consequently, periodic glutamine metabolism in cancer cells is altered. MYC disruption of BMAL1 can occur through upregulation of REV-ERBs, which inhibits the transcription of BMAL1, and/or through direct inhibition of BMAL1 transcription by binding to the promoter as an inhibitory MYC-MIZ complex (121, 122). It is noteworthy that these findings were determined in cancer cells with relatively low MYC levels and intact circadian rhythms. But like with HIF1α, high MYC levels and circadian oscillations are not incompatible since high levels of MYC, and intact oscillations have also been observed simultaneously in the same GBM cell line (13).

On the other hand, MYC is a clock-controlled gene itself. CRY2 can cooperatively bind MYC with FXBL3, promoting its ubiquitylation by SCF-FBXL3 and consequent degradation by the proteasome (123). This mechanism is also context-dependent because in mouse spleen, Cry2 knockout had no effect on Myc levels and Cry1/Cry2 double knockout repressed Myc levels (124). Per2 mutation and Bmal1 deletion in lung tumors has also been shown to lead to an increase in Myc levels, although specific detail is not clear (125). Another report using a mouse model suggests an indirect mechanism of Myc control by Bmal1 through catenin or Ctnnb1 as an intermediate effector (124).

### HIF-BMAL1 interactions

Another well studied pair of E-box binding proteins that have been shown to regulate each other is the bHLH-PAS TFs HIF1α and BMAL1. Under physiological conditions, two independent groups using different models showed that hypoxic responses mediated by HIF1α are under circadian control, and the hypoxic gene expression pattern is disrupted when BMAL1 is knocked out (126, 127). This is consistent with the presence of the E-box in the HIF1α promoter. In turn, HIF1α can participate in the regulation of circadian rhythm mediated by BMAL1 (127). Pharmacological stabilization of HIF results in a lengthened period and dampened amplitude of Per2 and BMAL1 rhythmicity. HIF1α also has a positive effect on the function of BMAL1 to activate transcription, as is shown in both studies (126, 127). Interestingly, BMAL1 and HIF1α can be co-immunoprecipitated in Co-IP experiments, suggesting that they can at least form a complex together or even dimerize to regulate gene transcription (127). ChIP-seq experiments of both factors in the same osteosarcoma cell line showed that BMAL1 and HIF1α share a large portion of binding sites on chromatin, which constitutes approximately a third of all HIF1α targets and a quarter of BMAL1 targets. These results further underscore their close relationship in regulating gene expression. However, their relationship is not well studied in cancer. Hypoxia-induced HIF activity is proposed to promote the disruption of the circadian rhythm in hepatocellular carcinoma, but a more detailed mechanism still needs to be revealed (128). Correlations between hypoxia/circadian clock and radiation resistance has been noted in glioma, yet mechanistic studies remain to be done (129).

Other regulators of the circadian clock, such as PER and CRY, also interact with HIFs but will not be elaborated upon here (130–132).

### EMT TFs interactions

The interactions between EMT-TFs and bHLH TFs are relatively less studied directly, although they share multiple phenotypic commonalities. The most understood interaction is with HIFs, which are mainly accounted for by their transcriptional regulation of each other and exemplified by the induction of EMT-TFs by HIF proteins. In multiple cell types, HIF overexpression or hypoxia is sufficient to induce EMT (133). HIF1α can directly bind to the HRE motifs in the promoter region of TWIST1, SNAI1, SNAI2 and ZEB1 to activate their expression. Indirect regulations of EMT-TFs by HIFs are often observed, too. For example, HIF1 can also activate histone modifiers such as HDAC3 to promote SNAI1 activation indirectly (134). Other intermediate genes include WDR5, lncRNA, FoxM1, ILK and PAFAH1B2 (135–137).

It is observed that high MYC levels and EMT often co-occur in cancer, and they can contribute to the same characteristics of later stage tumors. It has been reported that over-expression of MYC can induce EMT in lung cancer and melanoma cells through SNAI1 and ZEB (138, 139). MYC also facilitates TGFβ-induced EMT as a coactivator of the SMAD complex (140). This exemplifies physiological antagonistic factors can cooperate in certain tumors, because in physiological conditions, TGFβ represses MYC expression and inhibits cell proliferation, in turn MYC suppresses the activation of TGFβ-induced genes.

The interaction between circadian regulators and EMT factors has only been noted recently (141). It has been reported lately that the EMT process in cancer is gated by the circadian rhythm in the cell (142). Plus, BMAL1 has been shown to facilitate the EMT in colorectal cancer (143).

### Interactions within the same family

In addition to the inter-family mutual regulation, an intra-family interaction layer also exists. Most E-box-binding genes have some extent of redundancy. In physiological conditions, different members of the same family often share a large portion of target genes and implement similar functions in different tissues or at different developing stages. However, in cancer cells they often have independent or even antagonistic functions when they are simultaneously expressed in the same cells.

HIF family members provide prominent examples of such relationships (144). Pioneering work showed that in RCC cells where VHL function is defective, HIF2 fuels tumor growth partly by activating cyclin D1, and has suppressive interactions with HIF1 (145). In mouse xenograft models, HIF2 overexpression significantly enhanced tumor growth *in vivo*, whereas HIF1 overexpression suppressed tumor progression. Consistently, HIF1 overexpression lowered HIF2 protein levels, and vice versa. They also showed that the DNA binding function of HIF2 is responsible for its repressive activity towards HIF1 (145). The antagonistic functions of HIF1 and 2 are also marked in tumor associated stromal cells and have a reversed effect on angiogenesis in the tumor microenvironment (146). Despite all the examples, HIF1 and 2 are not always mutually antagonistic, but can also collaborate to meet different needs of the cancer cells (147).

Along similar lines, ZEB1 has been shown to participate in the initialization and progression of melanoma cells. By contrast, ZEB2 suppresses the onset and metastasis of melanoma in mouse (148, 149). SNAI1 and SNAI2 have also been shown to have opposite effects on the expression of phospholipase D (PLD), which has been proposed as a prognostic marker of breast cancer. PLD also has opposite effects on the expression of SNAI1 and SNAI2. The authors thus proposed a feedback loop model to explain the mutually antagonistic effect of the two factors on each other (150). In a different ovarian tumor model where SNAI1 and SNAI2 were also found to be mutually exclusive, SNAI1 was found to bind to E-boxes in the promoter region of SNAI2 and recruit HDAC to repress SNAI2 expression (151). More examples of EMT-TF mutual regulation can be found in ref (75).

All together, these examples show important features of the intimately connected EBTF network. First, the regulatory relationships among the TFs in normal tissues are largely rewired in cancer, and the new networks depend on tumor type and their evolutionary trajectory, thus are diverse. This explains the controversy that a certain EBTF is oncogenic in some tumor but is tumor-suppressive in others. Another feature is that in the rewired networks EBTFs are still closely related, because of their intrinsic DNA-binding specificity. On the one hand, targeting strategies on network levels might be needed to inhibit the tumor-fueling EBTF network. On the other hand, such network provides more actionable nodes to targeting certain EBTF or the whole network. Hypothetically, the flexibility of this network might also provide evolutionary spaces for tumor cells to develop plasticity.

## Perspectives of EBTFs in cancer

Because of the shared binding specificity and functional interconnectivity of the EBTFs, they also convergently regulate phenotypical hallmarks of tumors.

### Tumor initiation

Although all EBTFs discussed above are often dysregulated in cancer, they are rarely mutated (7, 9, 68), and the mechanism of how they “drive” tumorigenesis is still largely unknown. The rarity of their mutations implies the importance of the functional intactness of these proteins in cancer development.

MYC is the earliest and most documented oncogenic TF. Even a small disturbance of MYC homeostasis can induce abnormal phenotypic changes in cells (7). MYC can facilitate the progression of the cell cycle by upregulating genes that promote the passing of checkpoints. Interestingly, pan-cancer analysis showed that MYC amplification is mutually exclusive with many canonical oncogenic drives such as PIK3CA, PTEN, APC, and BRAF (7). This result implies that MYC has its specific mechanisms of driving tumorigenesis, likely through transcriptional regulation of the cell cycle.

Other EBTFs are not recognized as general cancer drivers so far in knock-out or over-expression-based *in vivo* tumor development assays. However, this does not exclude their potential oncogenic role since these models might miss some necessary background or cofactors, such as *de novo* enhancers gained through mutations in non-coding regions or epigenetic changes in the genome.

### Metabolism

Cancer cells usually require specific metabolic programs to meet their needs for continuous proliferation. The most prominent consequence of EBTFs in cancer is their ability to rewire the metabolic program of the cells, as a large proportion of metabolic genes contain E-boxes in their promoter region.

MYC, BMAL1-CLOCK and HIFs can all regulate genes that are responsible for glycolysis. Common gene targets include the GLUT family (114, 152), which controls glucose intake into the cell, and most of the enzymes involved in glycolysis. LDHA is also a well-documented gene regulated by MYC, HIF, and BMAL1 (153, 154). These enzymes together may cooperate to fuel the Warburg effect (155).

The TCA cycle turnover is also altered in cancer cells to support cancer progression. Prominently, MYC activates glutamine transporters and feeds more glutamine into the TCA cycle, resulting in the glutamine-addicted metabolic feature of many MYC-driven cancers (156, 157). MYC, HIFs, and BMAL1 can all regulate the source and level of acetyl Co-A entry into the TCA cycle and regulate lipid and cholesterol metabolism (114, 158, 159). Another pivotal TCA metabolite as a common gene target of EBTFs is α-KG, which is of great importance because it is a key node connecting metabolism with histone modification, marking the importance of epigenetic regulation by these factors, and with fatty acid synthesis through ACACA, which is also a shared target gene (159). It is also not surprising that all three factors can directly regulate the synthesis, elimination, and fusion dynamics of mitochondria (110, 160, 161). SREBP1, an essential regulator of cholesterol and fatty acid metabolism, has recently been shown to mediate circadian remodeling and maladaptive response to the over-nutritional environment of non-alcoholic fatty liver disease, which is a major risk of liver cancer (162).

It is noteworthy that BMAL1 and CLOCK are the master regulators of organismal metabolism in response to sleeping and feeding and coordinate metabolism across tissues and organs. This function is reviewed elsewhere (158).

### Immune evasion and inflammation

It is critical for tumor cells to evade the surveillance of the immune system, and during tumorigenesis malignant cells evolve multiple mechanisms to suppress the immune reaction against them. All the E-box binding proteins broadly participate in both innate and adaptive immune regulation (8, 163–165).

The recruitment of macrophages and other myeloid cells is the first level of immune regulation. MYC, HIFs, and BMAL1-CLOCK can all regulate cytokines responsible for their recruitment, including CCL family chemokines and interleukins. EBTFs can cooperate in a tumor to promote a conducive microenvironment. For example, MYC and TWIST have been shown to collaboratively support a pro-metastatic phenotype of macrophages through regulating the secretion of CCL2 and IL13 (166).

Immune checkpoint mediated by PD-L1 is another critical mechanism used by cancer cells to evade the immune response. MYC can regulate PD-L1 through either direct binding to its promoter or through post-transcriptional mechanisms in multiple types of cancers (167–169). PD-L1 is also reported to be a direct target of HIF1 (170), whereas BMAL1 regulates PD-L1 expression in an indirect way through lactate metabolism in macrophages (171). Regulation of PD-L1 has also been studied in EMT contexts (172).

NF-κB seems to be a central mediator of EBTF balance in immune regulation. MYC itself is a target of NF-κB (173). BMAL1 can dimerize with RelB and block a subunit of the NF-κB transcription complex (174). CLOCK can acetylate the RelA subunit and GRs to regulate their DNA binding activity (174, 175). Twist 1 can also interact with RELA (176).

### Angiogenesis and other tumor microenvironments

EBTFs also remodel other components of the tumor microenvironment, including extracellular matrix (ECM) components and promoting angiogenesis. Cancer-specific angiogenesis is an important feature of solid tumors and its potential as a therapeutic target has been underscored by the success of recent clinical trials involving anti-angiogenic therapy. VEGF is a central promoting factor of angiogenesis and has been shown to be directly regulated by HIF and BMAL1 (177). VEGF is also reported to be closely regulated by MYC (178, 179). BMAL1 has been shown to be associated with drug resistance of colorectal cancer cells via its regulation of VEGF (180). Other coordinating factors of angiogenesis have also been reported to be under EBTF control.

Recent advances in mechanobiology revealed the important role of ECM components and corresponding signaling pathways in tumorigenesis (181, 182). High ECM stiffness is a driving force of tumorigenesis and itself can result in an abnormal chromatin state (183). Collagen and integrin are the most studied ECM signaling-related molecules in cancer and have been shown to be regulated by EBTFs. The most prominent regulators are the EMT-TFs, which can directly regulate the type and amount of collagen genes produced by cells, and contribute to stem cell features of cancer cells (10, 184). MYC can regulate genes enriched in the ECM, cell adhesion and cell junction gene sets and regulate invasiveness (179). BMAL1-CLOCK has been reported to regulate the secretory pathway of collagens and maintain their homeostasis (185). HIFs also have well-documented functions in regulating ECM components by regulating collagen prolyl and lysyl hydroxylase and integrins (186).

### Cancer stem cells

Although the CSC concept still lacks a uniform definition across tumor types, some common features are recurrently observed in certain cancers such as glioblastoma, AML, breast cancer, HCC, etc. CSCs defined in these cancers usually have high heterogeneity, drug- and immune-resistance, and ability to self-renew. Interestingly, all the EBTFs discussed in this review are widely reported to be associated with CSCs (10, 12, 13, 187, 188). These examples imply that transcriptomic features might be able to uniformly define CSCs and guide targeting strategies.

**Figure 4:**
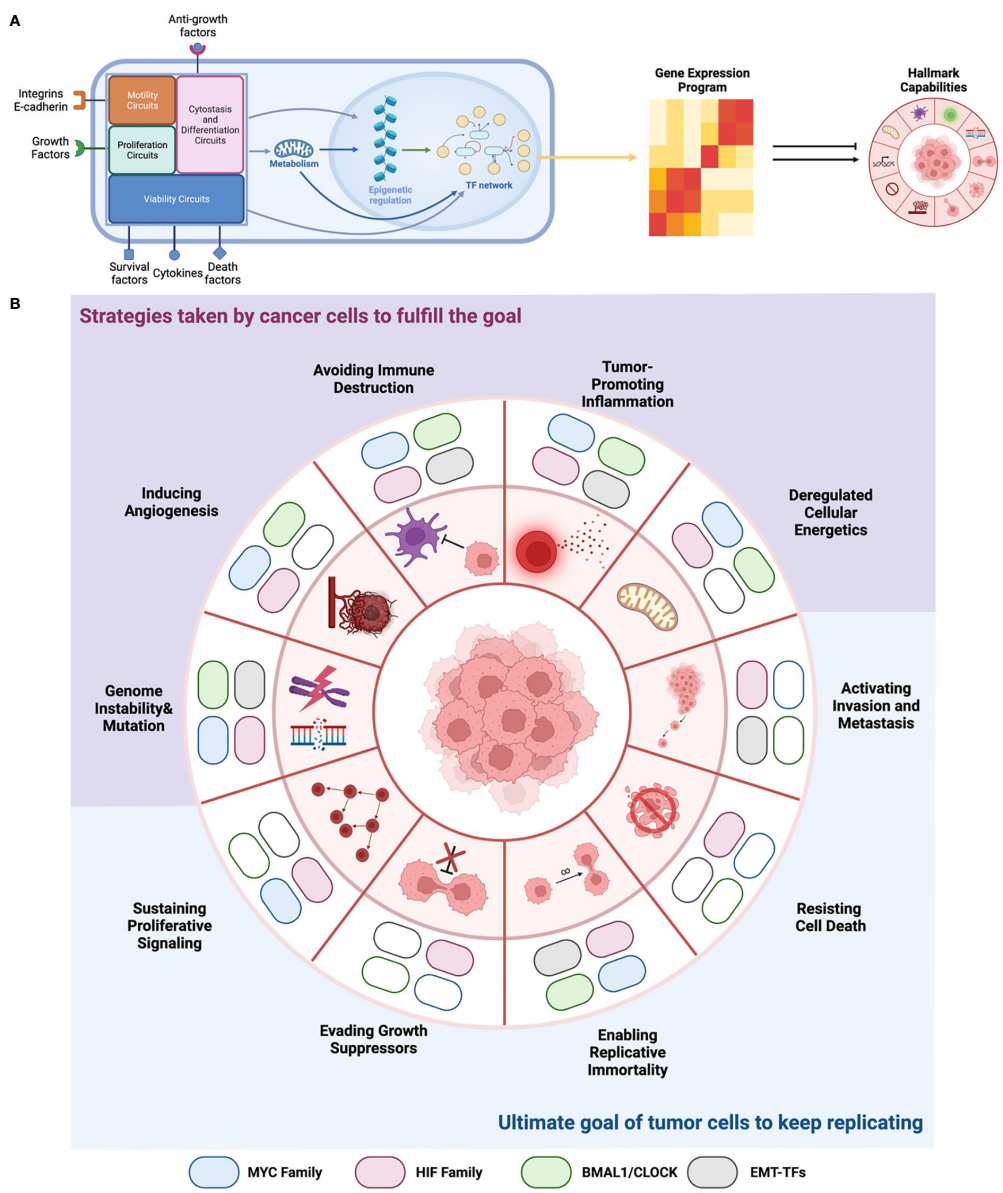
EBTFs Contributing to the hallmarks of cancer. **(A)** From a systems point of view, transcription factors are nodes that form the complex transcription regulatory network, which integrate all the information from external and internal signals. Then they decide which genes are transcribed and generate the gene expression program of the cell. The expressed genes then carry on the function to help tumor cells gain the hallmarks to progress. EBTFs form a subset of the whole network and carry out certain cellular functions. **(B)** Summary of EBTFs reported to contribute to the cancer hallmarks. Colored block indicates that the family is reported to contribute to the hallmark. Because the EBTF network regulates essential cellular activities, their function in tumors also contributes more to the pathological changes that cancer cells need to fulfill their ultimate goal of unceasing proliferation. Current results support the strategy of targeting the EBTFs to simultaneously eliminate the functional hallmarks thus halt tumor growth.

Summarizing all these functions, we propose a network perspective of E-box biology in cancer (**Figure 4A**) that bridges the fulfillment of phenotypic changes of cancer cells in different levels to meet their progressive needs. We also stratified their functions in line with the ten cancer hallmarks to highlight their specific involvements (**Figure 4B**).

## Targeting EBTFs in cancer

TFs was once thought to be “undruggable” targets because of their intrinsically disordered structure. But recent years new advances in pharmacochemistry provided promising new toolboxes for targeting TFs through various mechanisms such as inducing targeted protein degradation, disrupting protein-protein interaction, and indirect targeting of TF modulators and collaborators (189, 190).

MYC has been the most appealing yet challenging target among EBTFs for its centrality in cancer. The most prominent strategy is to target MYC-MAX dimerization. Early examples include OmoMyc, which is a 90 amino acid MYC mutant that bind to MYC and MAX to disrupt their dimerization (191). Initially OmoMyc was only thought of as a tool because of its size, but recently *in vivo* data showed its potential as a therapeutic (191). Small molecules that disrupt MYC function has also been successfully developed and show favorable pharmacokinetics and tolerability in preclinical studies (192). Multiple other mechanisms have also been explored, such as targeting MYC transcription, translation, and DNA-binding, etc. (193).

HIFs have attracted great interest as a therapeutic target in cancer for many years with multiple tested mechanisms (194). Recently, a small molecule named belzutifan that binds to the PAS-B domain of HIF2α showed exceptional efficacy in VHL-associated RCC in clinical trial (195). The results led to the first-in-kind approval from FDA to treat several cancer types associated with VHL. The success of belzutifan proved the feasibility of faithfully targeting bHLH-PAS TFs with small molecules and the therapeutic potential of these TFs.

Our lab and collaborators have developed several small-molecule-sets to target the circadian network and the activity of BMAL1 and CLOCK, including stabilizers of cryptochrome with precise isoform selectivity (196, 197), REV-ERB agonists (198), and novel inhibitors of casein kinase II (CK2) (199). We also showed the potential of these molecules as cancer therapeutics in pre-clinical models of multiple cancers (12, 13).

## Outlook

We progressively reviewed important EBTFs in cancer, their shared binding motif and target gene, close mutual regulation, convergent functions in homeostasis and cancer, and established a network view of the biology of these TFs. Synthesizing these factors in a unified model provide some important implications.

First, because EBTFs lie in a central node that fuels many hallmarks of cancer, targeting this node provides the chance of shutting down multiple hallmarks simultaneously. This is exemplified by the biology of MYC, which established a “coalition model” where MYC interacts with a wide variety of proteins, which cooperate to achieve a collaborative transcriptional program. Thus, it is proposed by the MYC-studying community that instead of targeting individual functions of MYC in different hallmarks, it is much more effective to “chop the MYC tree” to halt cancer cells from progressing (43).

On the other hand, however, this convergent view also imposes major challenges on studying the biology. First the functions of the fundamental connecting node, the E-boxes, remain largely unknown, making it hard to dissect the molecular mechanism of the EBTF network. It is also necessary to understand the determinants of the specificity of different EBTFs to different variants of E-boxes, such as DNA shape at the local motif (5), etc. This will provide a more balanced micro- and macroscopic perspective of EBTF biology in cancer. In addition, studying the properties of a network requires quantitative modeling, but acquiring data for establishing the model necessitates delicate experimental design. Luckily, well-characterized small molecules become available recently and provide handy tools for this purpose.

**Figure 5:**
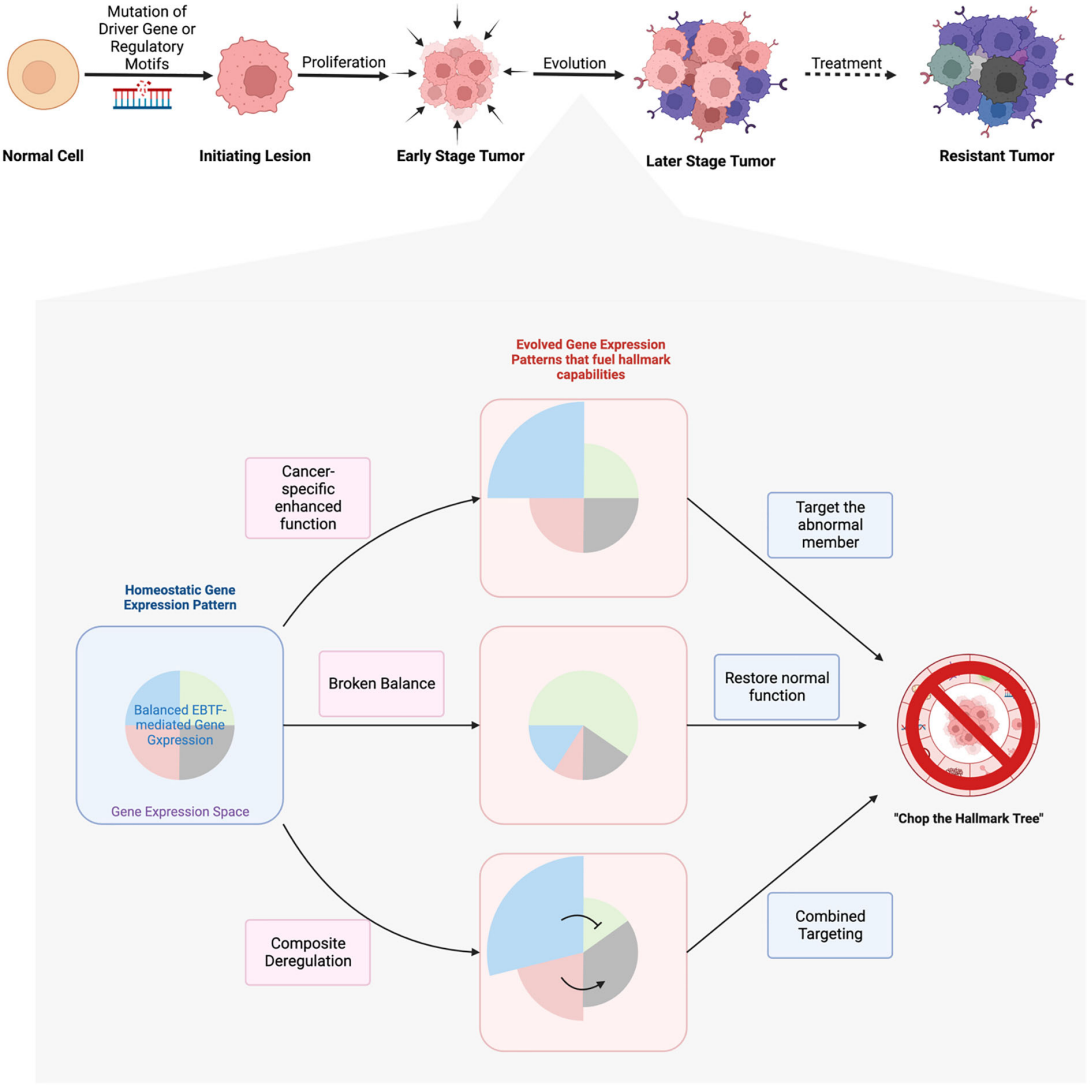
Hypotheses of EBTFs dysregulating transcription program during tumorigenesis and targeting strategies. The development of tumor is a long process from initial mutation to the evolved heterogenous tumor mass. Driver mutations in signaling molecules that sustain proliferation signal is the best understood mechanism of tumor initiation. In tumors that lack recurrent driver genes, mutations in the regulatory region of genes might be able to make up a transcriptional program to sustain a proliferation signal in the cell and initiate tumor. After initiation, tumor cells go through thorough evolution to grow into a tumor mass. Because transcription regulation is more flexible than obtaining new mutations, evolution of the transcriptome plays major role in the process of tumor development. In this process the transcriptional balance of TF networks such as EBTFs are broken in to promote the abnormal gene expression and fuel their progression. Such rewiring of the network can be caused by hyperfunction of one or more EBTFs through over-expression, *de novo* enhancers from mutations, and subsequent evolution of the transcriptome. To target these abnormal transcriptional programs, strategies can be taken to precisely eliminate hyperactivity of the master factors, restore function of factors that are suppressed by or competitive against oncogenic factors, or the combination of both.

Paving the ways for drugs that target EBTFs to clinic will be an important field of study. This will involve biomarker discovery for these targets, recognition of potential benefits, and careful design of pre-clinical and clinical trials, all of which requires better understanding of the biology of EBTFs. A promising first step would be combining EBTF-targeting drugs with current therapeutics. Because of the broad regulation of cancer hallmarks by EBTFs, there’s a higher chance that these drugs will synergize with current therapeutics to improve outcome. Successful examples include a MYC small molecule inhibitor, which is shown to synergize with anti-hormone therapy to inhibit prostate cancer and breast cancer cells (200), and several other studies that evaluated the efficacy of HIF inhibitors in combination therapies (**Figure 5**).

In summary, we depicted a network-based reasoning diagram for proposing and testing new hypotheses and strategies to target EBTFs in cancer. This model will help to account for the many discrepancies that have been encountered when trying to find a unifying function for one particular factor across different cancers. As chemical tools are becoming more available to regulate activities of EBTFs, we believe targeting EBTFs will be a promising new strategy for cancer therapy.

## Author contributions

YP and SK reviewed the literature and wrote the manuscript. PW contributed scientific insights of the topic and edited the manuscript. All authors contributed to the article and approved the submitted version.
